# Rare cause of a resistant hypertension in a middle‐aged man: A case report

**DOI:** 10.1002/ccr3.6606

**Published:** 2022-12-08

**Authors:** Renata Marecek, Eva De Keyzer, Georgiana Taujan, Felicia Baleanu, Mihaela Rosu, Ioanna Papadopoulou, Olga Kosmopoulou, Iconaru Laura

**Affiliations:** ^1^ Department of Internal Medicine, Centre Hospitalier Universitaire Brugmann Université Libre de Bruxelles Brussels Belgium; ^2^ Department of Cardiology, Centre Hospitalier Universitaire Brugmann Université Libre de Bruxelles Brussels Belgium; ^3^ Department of Endocrinology, Centre Hospitalier Universitaire Brugmann Université Libre de Bruxelles Brussels Belgium

**Keywords:** 11‐b‐hydroxylase deficiency, congenital adrenal hyperplasia, secondary hypertension, testicular adrenal rest tumors

## Abstract

Congenital adrenal hyperplasia associated to 11‐beta‐hydroxylase deficiency is a rare cause of secondary hypertension, usually discovered during childhood; however, a late diagnosis in adults has also been reported. Despite low cortisol levels, accumulated adrenal steroid precursors can activate the glucocorticoid receptor and thus protect the patient against adrenal crisis.

## Graphical abstract

Congenital adrenal hyperplasia due to 11‐beta‐hydroxylase deficiency might be a cause of secondary hypertension in late‐diagnosed adult patients.

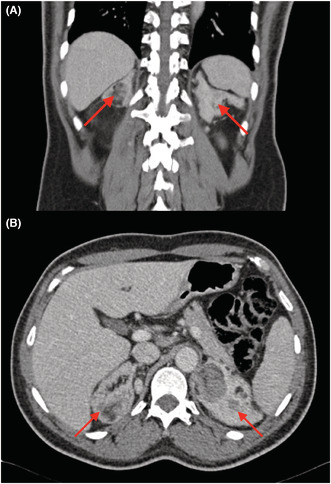

## 1 INTRODUCTION

Congenital adrenal hyperplasia (CAH) refers to a family of autosomal recessive disorders of adrenal steroidogenesis, in which each variant is characterized by a specific enzyme deficiency that impairs cortisol production by the adrenal cortex. The enzyme most commonly affected is 21‐ hydroxylase (21‐OH)^1^ followed by 11‐beta‐hydroxylase (11βOH), which accounts for 5%–8% cases of CAH, with an incidence of approximately 1:100,000.^2^

## 2 CASE PRESENTATION

A 43‐year‐old man was referred to the endocrinology department for the suspicion of secondary hypertension. The patient had a history of B‐cell non‐Hodgkin lymphoma and in remission for 13 years already after being treated with chemotherapy for 6 months. Moreover, he was diagnosed with arterial hypertension at the age of 5, for which he received bi‐therapy of carvedilol 6.25 mg twice daily and lisinopril 20 mg once a day.

**FIGURE 1:**
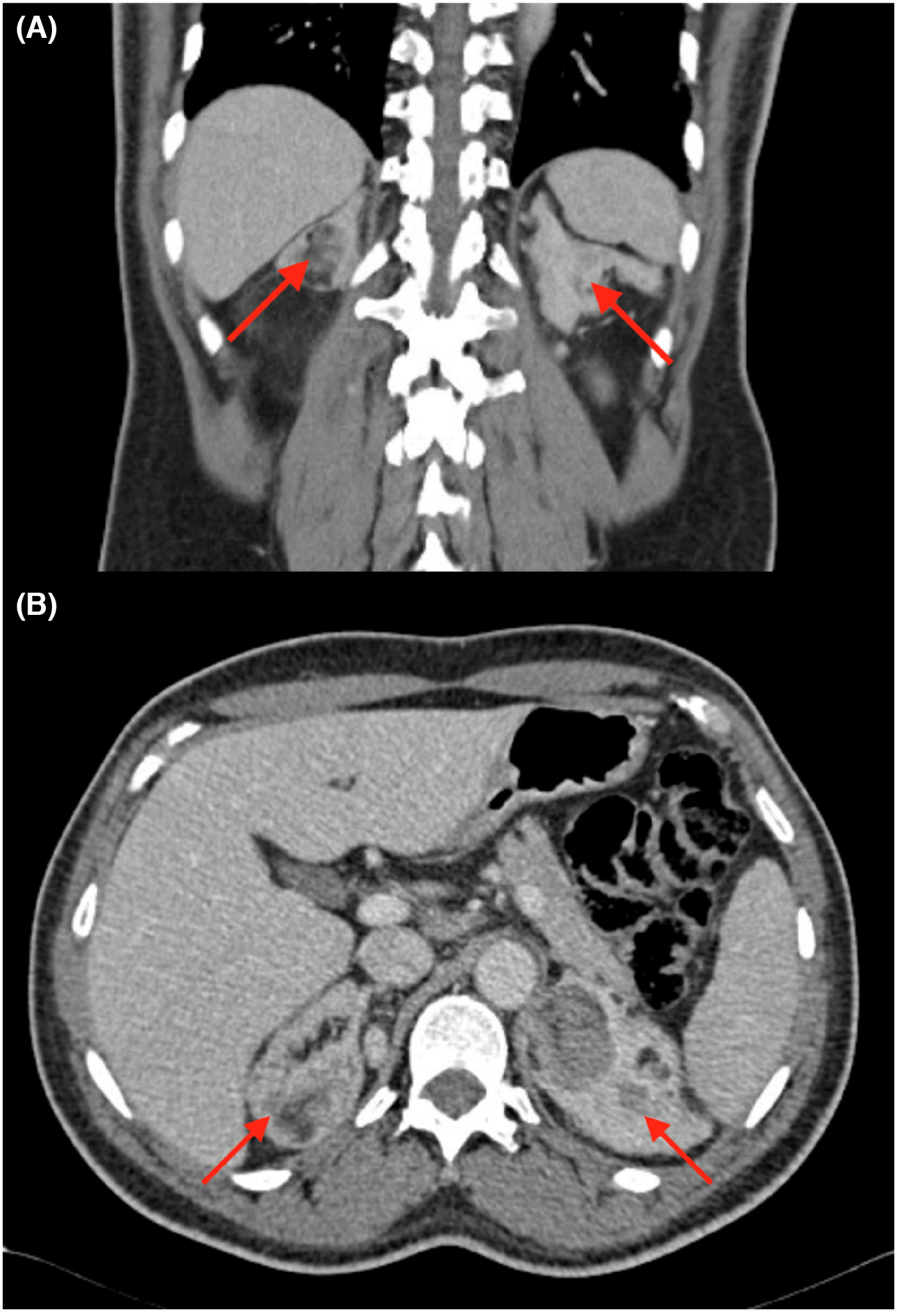
On the abdominal computed tomography (A: coronal view, B: axial view), the red arrows show a bilateral enlarged adrenal glands associated with voluminous lesions with lipomatous density

**FIGURE 2:**
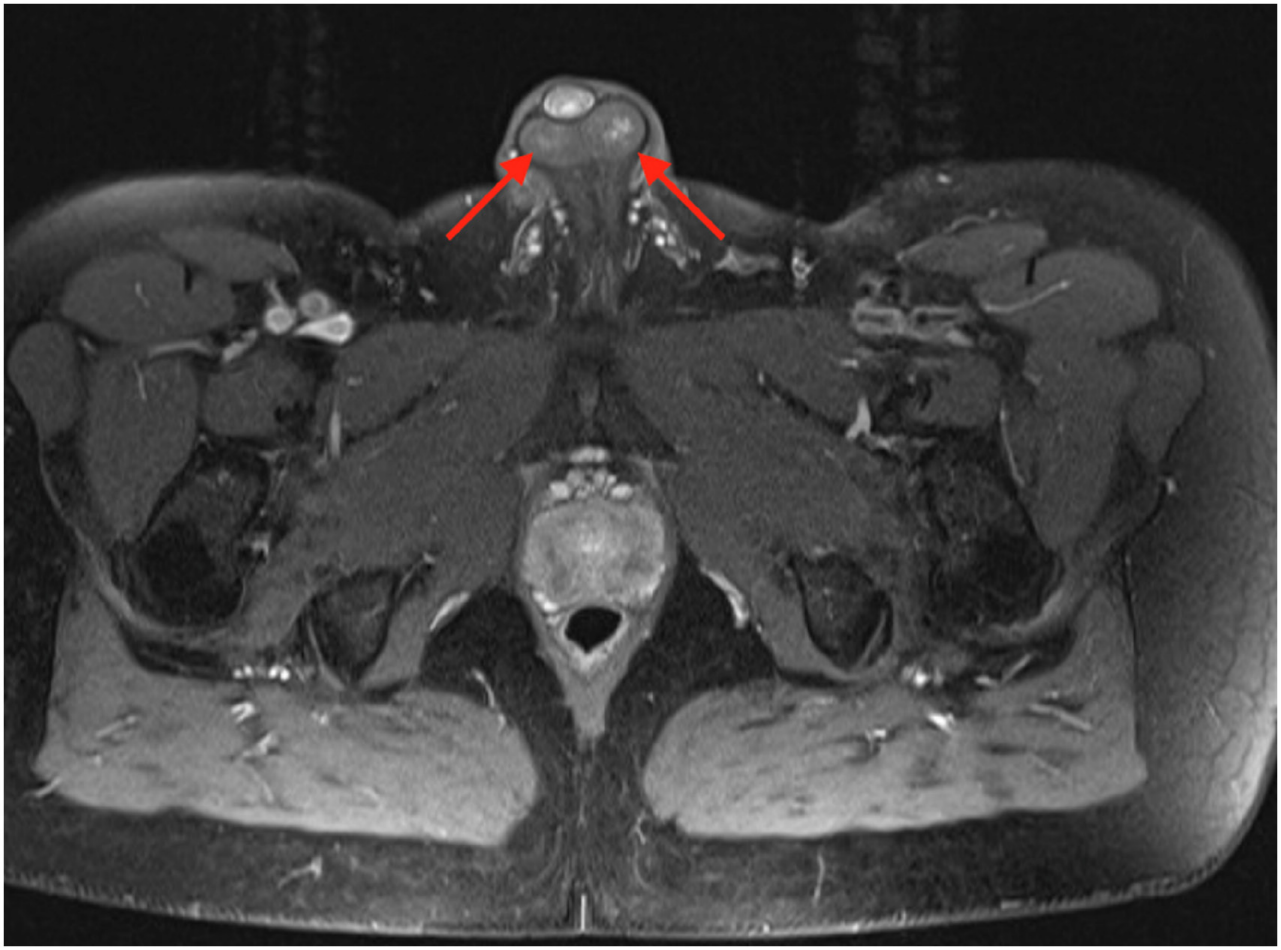
The axial view of scrotal magnetic resonance imaging: the red arrows show a bilateral intratesticular lesion compatible with an adrenal intra testicular inclusion

For the last 2 years before presentation, the patient associated dyspnea on exertion (NYHA II). The patient has a history of hypertension in the family. At medical consultation, he reported premature adrenarche with an early pubic hair development. The physical examination revealed short stature (158 cm) and a hypertension with a blood pressure (BP) of 190/120 mmHg. The echocardiography showed a moderate left ventricle concentric hypertrophy, with an ejection fraction of 40%, with no valvulopathy nor dyskinesia. The laboratory results (Table 1) showed a negligible hypokalemia at 3.4 mmoL/L (reference value: 3.5–4.5 mmoL/L), an elevated ACTH level of 921 pg/ml (reference value: 6–60 pg/ml), with low cortisol level at 117 mmoL/L (reference value: 166–507 mmoL/L), normal renin level at 9 μUI/ml (reference value: 4.4–46.1 μUI/ml), and low‐normal aldosterone level at 22.8 ng/L (reference value: 22.1–353 ng/L). Further investigations demonstrated a minimal elevation of the testosterone level at 30.4 nmoL/L (reference value: 8.64–29 nmoL/L) and elevated values of dehydroepiandrosterone (DHEAS) at 17.8 μmoL/L (reference value: 2.4–11.6 μmoL/L), androstendione at 31 ng/ml (reference value <3 ng/ml, 17–hydroxyprogesterone (17OHP) at 8 ng/ml (reference value: 0.9–3.4 ng/ml), and 11‐DC at 30 ng/ml (reference value <0.5 ng/ml). The results were compatible with CAH due to 11βOH deficiency. The patient confirmed the absence of adrenal insufficiency crisis until the moment of presentation. The abdominal scanner showed bilateral enlarged adrenal glands with voluminous lesions with lipomatous density (right side: 66 × 53 × 88 mm and left side: 55 × 40 × 52 mm) (Figure 1). The scrotal ultrasound and MRI showed bilateral intratesticular lesions compatible with adrenal intratesticular inclusion (Figure 2). The genetic tests revealed a homozygote pathogenic variant of the gene CYP11B1, chromosome 8, exon 8, protein pArg448His.

**TABLE 1:** Laboratory values before and after treatment

	Normal values	Before treatment	After treatment
Cortisol	166–507 mmoL/L	117 mmoL/L	14 mmoL/L
ACTH	6–60 pg/ml	921 pg/ml	38 pg/ml
11‐Deoxycortisol	<0.5 ng/ml	30 ng/ml	2.5 ng/ml
Potassium	3.5–4.5 mmoL/L	3.4 mmoL/L	4.2 mmoL/L
Renin	4.4–46.1 μUI/ml	9 μUI/ml	8.6 μUI/ml
Aldosterone	22.1–353 ng/L	22.8 ng/L	<19.1 ng/L
17‐Hydroxyprogesterone	0.9–3.4 ng/ml	17.8 μmoL/L	2.1 ng/ml
Testosterone	8.64–29 nmoL/L	30.4 nmoL/L	14.3 nmoL/L
Androstendione	<3 ng/ml	31 ng/ml	5.3 ng/ml
Dehydroepiandrostendione	2.4–11.6 μmoL/L	17.8 μmoL/L	6.08 μmoL/L

Treatment by dexamethasone 0.5 mg once per day was initiated with the improvement of laboratory results after 2 months (the ACTH level decreased at 38 pg/ml), with a low cortisol level of 14 mmoL/L, potassium level normalized (4.2 mmoL/L), and all the androgens and adrenal precursors decreased to values in reference ranges: testosterone, 14.3 nmoL/L; androstendione, 5.3 ng/ml; DHEAS, 6.08 μmoL/L; 17OHP, 2.1 ng/ml; 11‐DC 2.5 ng/ml (Table 1). At the 4 years follow‐up, his BP was normalized at 128/84 mmHg under monotherapy (lecarnidipine 20 mg per day). The echocardiography showed normalization of the left ventricle ejection fraction. The testicular ultrasound demonstrated a relative decrease in volume of the testicular lesions. The patient refuses the testicular biopsy.

## 3 DISCUSSIONS

The 11βOH deficiency accounts for 5%–8% of patients with CAH.^2^ The patients are usually diagnosed before the age of 13 years^2^, ^3^, ^4^, ^5^ and mostly before the age of 4 years.^3^ Late diagnosis in adult aged 28 years and above has been reported; however, it is rare.^6^, ^7^, ^8^

**FIGURE 3:**
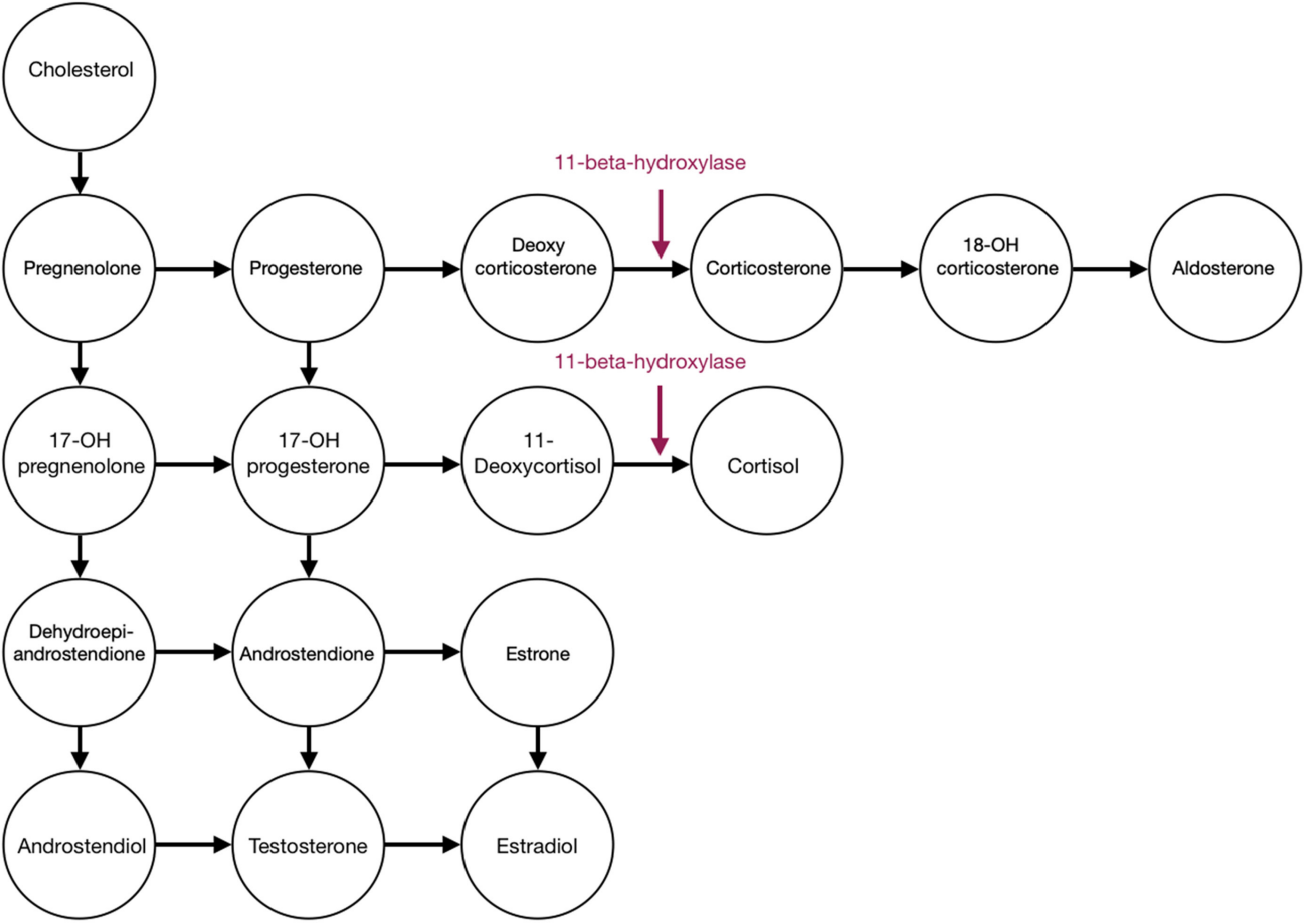
The adrenal steroidogenesis pathway

The 11βOH converts 11‐desoxycortisol (11‐DC) to cortisol and 11‐deoxycorticosterone (DOC) to corticosterone.^9^ The decreased cortisol secretion results in elevated adrenocorticotropic hormone (ACTH) plasma level as well as an overproduction of steroid precursors and androgens (see the adrenal steroidogenesis pathway described in Figure 3). Androgen excess produces virilization and precocious pseudo‐puberty. The mineralocorticoid effect of the elevated DOC can lead to hypertension in up to two‐thirds of untreated patients.^10^ Excessive ACTH production results in hyperplasia of ACTH‐sensitive tissues in adrenal glands and others sites such as the testes, causing adrenal masses and testicular masses known as testicular adrenal rest tumors (TARTs).^11^

The 11βOH deficiency occurs as a classic or non‐classic phenotype, depending on the degree of clinical severity and the percentage loss of enzyme activity.^3^ Non‐classic phenotype presents no abnormalities at birth and later can present mild virilization, peripheral precocious puberty, with premature adrenarche and penis enlargement in boys, and hirsutism as well as menstrual irregularities in girls.^12^ Patients with non‐classic 11βOH deficiency usually do not develop hypertension.^6^ Untreated patients may present accelerated skeletal maturation, resulting in short final stature.^3^ The clinical signs are usually more evident in female patients than in males.

Engels et al. reported that the accumulated adrenal steroid precursors in patients with CAH can activate the glucocorticoid receptor and protect the patients against adrenal crisis.^13^ The most potent glucocorticoid receptor activating steroid precursors are 11‐beta‐hydroxylated steroids ‐ 11‐DC and DOC.^14^, ^15^ This mechanism that could explain the late diagnosis present in several rare cases described in the literature. The non‐inclusion of measurement of 11‐DC in dry blood spots in neonatal screening programs and the presence of elevated 17OHP that may lead to the misdiagnosis of 21‐OH deficiency are other two factors that can contribute to a delay in diagnosis.

Arterial hypertension is another important feature of the classic form of this deficiency. The exact pathophysiology of hypertension in 11βOH deficiency remains unclear. Peter et al. discussed the predominant role of the DOC with intrinsic mineralocorticoid activity as a possible cause.^15^ However, the DOC levels do not correlate with the severity of hypertension.^6^ Overall, the hypertension in 11βOH deficiency is hyporeninemic, without overt alterations in serum potassium and sodium concentrations.^16^ Breil et al. reported several cardiovascular conditions associated with 11βOH deficiency such as left ventricular hypertrophy, ischemic heart disease, hypertensive retinopathy, and cerebrovascular accidents^2^, and some studies have demonstrated that the left ventricular hypertrophy in 11βOH deficiency can be reversed after bilateral adrenalectomy.^17^, ^18^

The intra‐testicular inclusions are present in approximately 42% of cases of male patients with CAH.^19^ The tumor growth increases the intra‐testicular pressure and reduces blood flow causing testicular damage with resulting oligo‐ or azoospermia.^2^ Despite their benign character, monitoring of TARTs is important as they are hardly distinguishable from the Leydig‐cell tumors (LCT). Bilateral tumors are more frequently seen in TARTs with 83% of cases than LCT with only 2.5% of cases.^20^ A testicular biopsy can always be performed to help in the differential diagnosis. These inclusions are ACTH‐dependent benign tumors and can regress with ACTH suppression in most cases.

The treatment modalities in 11βOH deficiency consist of glucocorticoid suppressive therapy and surgical correction of the ambiguous external genitalia in virilized female patients.^17^ The glucocorticoids can substitute for the cortisol deficiency and inhibit ACTH oversecretion and thus suppress the excessive androgen and mineralocorticoid production. However, in CAH, an effective suppression of ACTH sometimes requires high doses of glucocorticoid over a prolonged period of time,^21^ which explains the difficulty to maintain a satisfactory adrenal suppression without producing an unacceptable degree of hypercortisolism. The bilateral adrenalectomy was proposed as an alternative; nevertheless, the patient compliance is required for a lifelong hormonal substitution. Nasir et al. opted for it in the management of a difficult case with failure to suppress androgen production.^22^ Chabre et al. have applied it in a case of a patient with severe hypertension who had experienced long‐term difficulties with equilibrium and compliance with the suppressive therapy.^17^ Finally, Hinz et al. reported bilateral adrenalectomy in a 15‐year‐old patient with resistant hypertension despite good compliance.^23^

## 4 CONCLUSIONS

The 11βOH deficiency is a pediatric pathology, which can be rarely diagnosed even in adulthood. The diagnosis can be delayed due to a poor clinical presentation, especially in men, and the fact that the patients with this form of congenital adrenal hyperplasia do not usually develop adrenal crisis. Once the diagnosis was established, male patients should be screened for testicular adrenal inclusions.

Despite the late diagnosis and the long evolution of arterial hypertension, our patient had a good therapeutic response once the glucocorticoid suppressive therapy was started, with a better control of arterial pressure by monotherapy only, normalization of left ventricular ejection fraction, normalization of ACTH, androgens, and adrenal precursors, but with only a minimal decrease in volume of the testicular adrenal rest tumors.

## AUTHOR CONTRIBUTIONS

RM: wrote the first draft of the manuscript. LI, FB, ED, GT, IP, OK, MR: revised subsequent versions of the manuscript. All authors read and approved the final version of the paper. RM accepts responsibility for the integrity of the data analyses.

## CONFLICT OF INTEREST

All authors state that they have no conflicts of interest.

## ETHICAL APPROVAL

The consent has been obtained from patient after full explanation of the purpose and nature of all procedures used.

## CONSENT

Written informed consent was obtained from the patient to participate in this study for the publication of this case report. A copy of the written consent form is available for review on request.

## Data Availability

Data are available for review on request.
